# Time-Resolved Observation of the Destination of Microinjected Potato Spindle Tuber Viroid (PSTVd) in the Abaxial Leaf Epidermal Cells of *Nicotiana benthamiana*

**DOI:** 10.3390/microorganisms8122044

**Published:** 2020-12-20

**Authors:** Hyesu Seo, Ying Wang, Woong June Park

**Affiliations:** 1 Department of Molecular Biology, Dankook University, Chonan-si, Chungnam 31116, Korea; hsuseo89@daum.net; 2 Department of Biological Sciences, Mississippi State University, Starkville, MS 39762, USA; wang@biology.msstate.edu

**Keywords:** potato spindle tuber viroid (PSTVd), nuclear import, microinjection, *Nicotiana benthamiana*

## Abstract

Viroids are single-stranded noncoding RNA molecules of 250–400 nucleotides that cause plant diseases. One of the two families of viroids is *Pospiviroidae,* the members of which replicate in the nuclei of host cells. To replicate in plants, viroids of *Pospiviroidae* must enter the nucleus. However, the nuclear import of viroids remains understudied. In this work, we documented the time-dependent characteristics of the changes in microinjected fluorescently labeled potato spindle tuber viroid (PSTVd). The cytoplasmic fluorescence disappeared gradually, with only nuclear fluorescence remaining as the PSTVd injected in the cytoplasm was imported into the nucleus. Through this work, we determined that the time for half-maximal nuclear accumulation of the viroid was about 23 min. Interestingly, we found some cells where the nuclear import did not occur, despite the high level of cytosolic viroid injected. In some cells, the injected viroids disappeared within 10–20 min. The nuclear import of PSTVd is not a simple concentration-dependent process but was probably under the regulation of diverse factors that may be missing from some cells used for our observation.

## 1. Introduction

Viroids are small (250–400 nucleotides), noncoding single-stranded RNA molecules [1,2] that proliferate and move to other plant organs once they successfully infect host plants [3]. Generally, viroids are classified into two major families: *Pospiviroidae* and *Avsunviroidae* [4]. Viroid species of the *Pospiviroidae* family replicate in their host cells’ nuclei using the plant RNA polymerase II [5]. Initially, some viroids of *Pospiviroidae*, such as potato spindle tuber viroid (PSTVd) [6,7] and citrus exocortis viroid (CEVd) [8,9], were recovered from the isolated nuclei of viroid-infected plant leaves, suggesting that the viroids exist in plant nuclei. Later, high-resolution in situ imaging confirmed the intranuclear localization of viroids, for example, PSTVd [10], CEVd, and coconut cadang cadang viroid [11] in plants.

To replicate in plant cells, viroids of *Pospiviroidae* must enter the nucleus. However, besides several reports, the nuclear import of viroids has received little attention. Harders et al. [10] demonstrated the intranuclear localization of PSTVd in viroid-infected tomato leaves by in situ hybridization and confocal microscopy. However, these investigation techniques did not distinguish the imported PSTVd from the newly replicated viroids. Although advanced fluorescence in situ hybridization techniques combined with high-resolution microscopy verified even the strand-specific localization of PSTVd in the nucleoplasm and nucleolus [12], the imported and replicated viroids were undistinguished. In vitro experiments on permeabilized protoplasts described the nuclear import of PSTVd as saturable and specific [13]. It was the first report to determine the time-dependent characteristics of the nuclear import of PSTVd; PSTVd was detected in 20–30% cells at 20 min and in 70% at 40 min [13]. The same paper also noted that the nuclear import of PSTVd was independent of cytoskeleton and Ran GTPase cycles in protoplasts [13]. Microinjection is a good choice to investigate the time-dependent changes in nuclear import and cytoplasmic clearance. When applied to tobacco leaves, nuclear import of fluorescently labeled PSTVd was revealed 10 min after injection [14]. However, the authors focused on the cell-to-cell trafficking of PSTVd and did not follow up on the nuclear import.

In this current work, we observed the early events of nuclear import of PSTVd after microinjection. For close monitoring of the nuclear import of PSTVd, we used abaxial epidermal strips of *Nicotiana benthamiana,* which maintained its original cell shape. We present time-course images of the cells injected with fluorescently labeled PSTVd and discuss several possibilities to explain the destination of the injected PSTVd.

## 2. Materials and Methods

Seeds of *N. benthamiana* were soaked and spread on soil in the small pots. The seedlings were grown in a growth chamber kept at 16-h-light/8-h-dark (28 °C) cycles. Young leaves less than 1 cm in length were collected from 10- to 15-day-old plants. The abaxial epidermis was peeled off in accordance with the protocol for preparing protoplasts [15]. We collected the thin, transparent, sticky tape with attached abaxial epidermal strips that floated in distilled water.

PSTVd (PSTVd-intermediate) was labeled with Alexa Fluor 488 (AF488; green fluorescence) or Alexa Fluor 546 (AF546; red fluorescence) by in vitro transcription using the MAXIscript transcription kit (Ambion, Austin, TX, USA) according to the manufacturer’s protocols in the presence of AF488-5-UTP or AF546-5-UTP (Molecular Probes, Waltham, MA, USA). We used 1-µg *Hin*dIII-linearized pGEM-T vector-based PSTVd clones as the templates for AF546-labeling in vitro transcription with T7 polymerase. pDONR221-T3-PSTVd linearized by *Sma*I was used for AF488 labeling with T3 polymerase [16]. The PSTVd clones were kindly donated by Biao Ding (Ohio State University, Columbus, OH, USA). The labeled PSTVd was purified with NucAway Spin Columns (Ambion) to remove unincorporated fluorescent material. Finally, the solution for microinjection was prepared in an Eppendorf tube: 3 µL of purified Alexa Fluor-labeled PSTVd, 3 µL of 4′,6-diamidino-2-phenylindole (DAPI; 25 ng/µL), and 7 µL of distilled water.

For microinjection, the abaxial epidermal strip was placed on a glass slide with the transparent tape faced down and the cells were exposed. Some drops of distilled water were supplied to prevent the cells from drying out. The injection needle, which was made of a glass capillary with a needle puller (PC-10, Narishige, Tokyo, Japan), was put in the Eppendorf tube to be filled with the injection solution by capillary action. The needle containing the solution was then connected to the manual microinjection system (Narishige, Tokyo, Japan) attached to a Nikon E600 fluorescence microscope (Nikon, Tokyo, Japan). After focusing the cells and the injection needle, the needle’s position was finely adjusted and the fluorescently labeled PSTVd-containing solution was injected. The needle was removed immediately after the injection, and a cover glass was put on the epidermal strip. The corners of the cover glass were supported with small drops of modeling clay, taking care not to press the cells too much. By using a long-focus objective lens (Nikon, Tokyo, Japan), we could make space between the glass slide and the lens to operate the microinjection system. The fluorescence images were captured time-dependently. To quantify the fluorescence signals from the nuclei, we used a public-domain software ImageJ (https://imagej.nih.gov/ij/). We separated the color channels of each image and then measured the proper signals.

## 3. Results and Discussion

**Figure 1:**
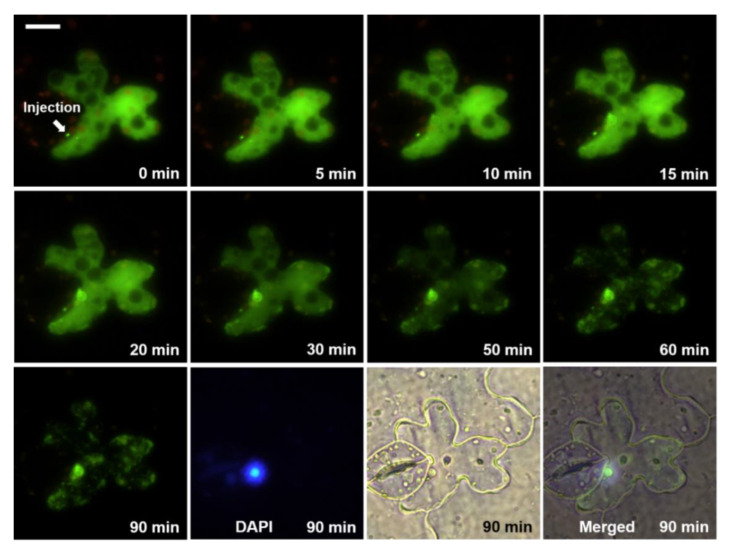
Time-course images of an abaxial leaf epidermal cell after microinjection of AF-488-labeled potato spindle tuber viroid (PSTVd; green): DAPI (blue) shows the nucleus and the white bar indicates 20 µm. These images and similar photos are also presented in Video S1.

We microinjected PSTVd into the abaxial leaf epidermal cells of *N. benthamiana* to monitor the nuclear import and other changes following the injection. For real-time observation of the PSTVd changes, we labeled PSTVd with AF488 or AF546 to produce green or red fluorescence, respectively. When the Alexa Fluor-labeled PSTVd was introduced into the cell by microinjection, the labeled PSTVd spread in the cytosol, except for leaving some dark spots, which may be membrane-enclosed intracellular compartments (Figure 1). This result supports the idea that PSTVd did not cross the biological membrane by passive transverse mode, which is the prerequisite for selective import of viroids into the nucleus or chloroplast, as reported previously [1,2].

**Figure 2:**
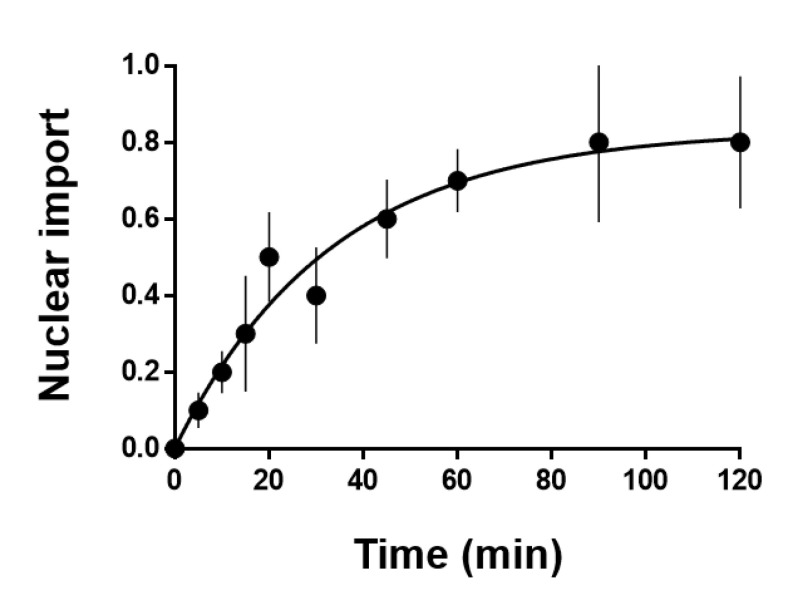
Time-dependent nuclear import of PSTVd after microinjection: fluorescence signals were measured and expressed as relative changes form the initial (baseline) level and then normalized. Data are shown as mean ± standard error (vertical bars). *n* = 7, 9, 9, 9, 9, 8, 10, 4, and 4 serially from 5 to 120 min. This plot was prepared using a software, Prism 6.0 (GraphPad Software, San Diego, CA, USA).

The nuclear accumulation of the labeled PSTVd was initially invisible. However, after a specific duration, the nuclear fluorescence from PSTVd intensified with time (Figure 1 and Video S1). The position of the nucleus was confirmed by the DAPI signal. We attempted to determine the time required for half-saturation of the viroidal nuclear import. However, simple plots of the measured fluorescence intensity did not show a clear time-dependent nuclear import pattern of the injected PSTVd because the fluorescence signals from the investigated cells were highly variable. Variations in the cell size, physiological conditions, incorrectly controlled injection volume, and many other factors could cause this inconsistency. Therefore, it was not feasible to decipher the time-dependent characteristics of the nuclear import of PSTVd using absolute values. To overcome this complexity, we expressed the fluor1escence intensity relative to the baseline (initial level) measured immediately after the microinjection. Then, the values obtained from the same nucleus at different times were normalized. Plots based on the normalized values against the time after microinjection revealed a saturable curve (y = 0.830875exp(−0.02997x) + 0.0009625) with the half-maximum value at about 23 min (Figure 2). Because the fluorescence signal from some samples reached maximum at 90 min and decreased again, the maximal value did not reach the expected 1.0 but remained at 0.8 at 120 min.

The saturable mode of the nuclear import indicates the existence of saturable carriers or binding factors supporting Woo et al. [13], who stated that the nuclear import of viroids are competitive and specific. A close look at the cell at 5 or 10 min after injection with AF488-labeled PSTVd (Figure 1) revealed a green, fluorescent ring where the nucleus was stained with DAPI. PSTVd probably attached first to the nuclear membrane before passing it through nuclear pores. Afterward, the fluorescence signal in the nucleoplasm intensified with time (Figure 1 and Video S1). In some cases, fluorescence signals from Alexa-labeled PSTVd were concentrated at certain positions in the plant nucleus (Video S1A,B,D), similar to the images that illustrated localization of PSTVd in the nucleoli [12]. The fluorescence signal from the AF488-labeld PSTVd even shone with speckled patterns in the nucleus in some cells (Video S1C,E).

**Figure 3:**
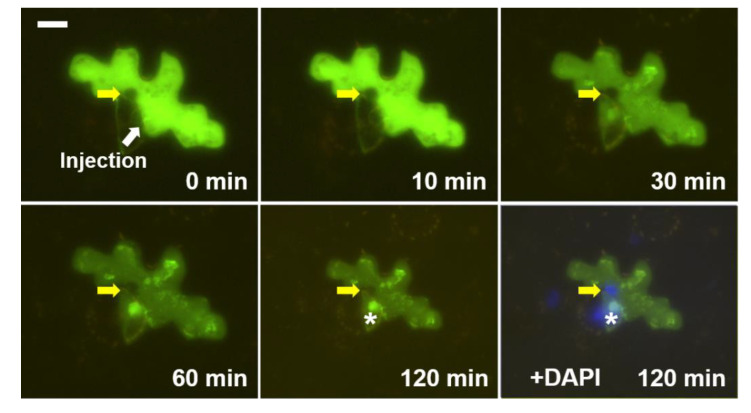
Example of microinjected cells showing no nuclear import of PSTVd despite of high level of cytosolic viroid injection: AF488-labeled PSTVd looks green, DAPI (blue) shows the nucleus, yellow arrows point the nucleus of the injected cell, asterisks (*) indicate the nucleus of the neighboring guard cell and the white scale bar is 20 µm. These photos and similar images are also presented in Video S2.

The nuclear import of AF488- or AF546-labeled PSTVd was well documented. However, despite the high accumulation of PSTVd in the cytosol, there was no nuclear import in some cases (Figure 3 and Video S2). In Figure 3, the injected AF488-labeled PSTVd did not enter the nucleus of the epidermal cell; the site of the nucleus appeared as a dark spot (yellow arrows).

However, interestingly, the neighboring guard cell, which was not intentionally injected but slowly gained the labeled PSTVd probably because of leakage of the AF488-labeled PSTVd at the injection site, showed distinct nuclear import (Figure 3, asterisk). It seems that the unique biological condition of each cell can be utilized by the injected viroids.

These results suggest that the nuclear import of viroids is not simply concentration-dependent but occurs only when certain biological conditions are fulfilled. Even in the cells where we observed nuclear import of PSTVd, the efficiency of the nuclear import did not necessarily correlate with the level of viroids in the cytoplasm. As diverse protein factors are involved in the nuclear import of proteins [17], some cytosolic factors may contribute to the nuclear import of viroids. Some viroid-binding proteins that have nuclear localization signals have been reported [16,18,19,20]. Although their roles during the nuclear import of viroids remain unillustrated, the contribution of the previous reported or yet unknown factors could be reasonably postulated.

**Figure 4:**
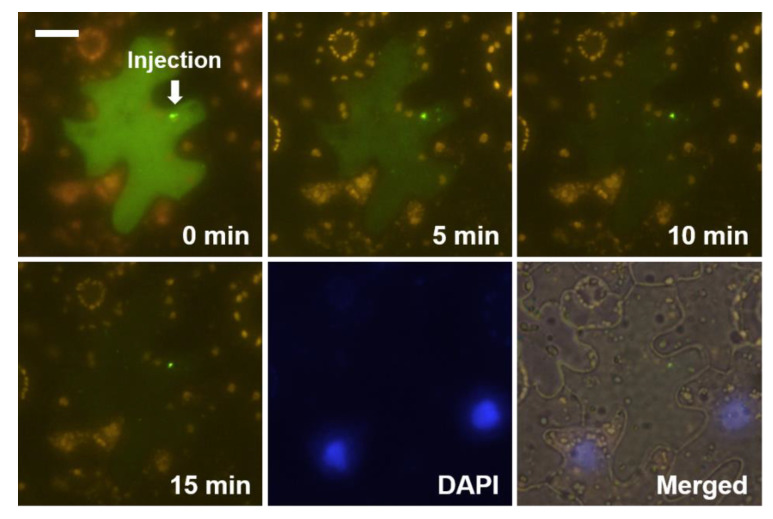
Rapid disappearance of the injected PSTVd (green) observed in some cells without nuclear import: DAPI (blue) shows the nucleus and the scale bar indicates 20 µm.

The microinjected PSTVd disappeared rapidly from the cytosol in some cells (Figure 4). In such cases, we failed to stain the nucleus quite often. The absence of a DAPI signal in the injected cells was unexpected because we added DAPI in the injection solution and observed DAPI-stained nuclei in neighboring cells. The exact cause of this phenomenon remains unverified. Viroids are trafficked through plasmodesmata [14] from the epidermis to palisade mesophyll in *N. benthamiana* [21]. The fast disappearance of the injected PSTVd may be due to vigorous trafficking of the viroid. If the trafficking is not molecular action-based but somewhat bulky and fast enough, the flow of DAPI in the same solution could be affected.

The level of labeled PSTVd in the cytoplasm of the injected cells decreased gradually, even when we observed nuclear import in the same cell. The rate of decrease was quite diverse (Figure 1, Figure 3 and Figure 4) depending on the cells, indicating cell-autonomous responses to the injected viroids. These observations provided insights into the turnover process of viroid RNA separate from the replication. It is intuitive to speculate the involvement of host RNA silencing machinery. Cytosol-localized dicer-like 2 protein [22], which has been shown to play a role in defending viroid infection [23,24], may participate in this cytoplasmic clearance process. However, we cannot rule out the involvement of other host ribonucleases. Besides observing that the injected PSTVd was degraded and transported, the PSTVd appeared aggregated in the cytoplasm (Figure 1 and Figure 3). The nature of the aggregate-like spots and distribution patterns of PSTVd observed in the cytoplasm remain to be explored.

## Supplementary Materials

The following are available online at https://www.mdpi.com/2076-2607/8/12/2044/s1, Video S1: Nuclear accumulation of fluorescently labeled PSTVd after microinjection for (A–E) AF488 and (F,G) AF546, Video S2: Examples showing no nuclear import of the injected viroid despite a high cytoplasmic level of PSTVd for (A) AF488 and (B) AG546.
